# On the Constitution and Source of the Bile

**Published:** 1864-03

**Authors:** Thomas Antisell

**Affiliations:** Surgeon U.S.V., Professor of Physiology and Military Surgery in the Medical Department of Georgetown College, D.C.


### ON THE CONSTITUTION AND SOURCE OF THE BILE.

By THOMAS ANTISELL, Surgeon U.S.V., Professor of Physiology and Mil. Surgery in the Medical Department of Georgetown College, D.C.

During the autumn of the last year and the past spring, having had the opportunity of making *post mortem* examinations of patients who had died of what is commonly known as chronic diarrhœa, and finding the gall-bladder (as might be expected in such subjects) distended with bile, it appeared to me that the application of dialysis to this liquid might determine something new regarding either its constitution or uses, and go to settle some of the obscure and doubtful points in its natural history. It must be admitted that this mode of dialysis has much to recommend it as a means of investigation of animal liquids, for it permits of the examination of the animal fluid while yet in a fresh state, and by using the microscope in connection, enables us to observe the substance itself by the eye, and recognize the form. Nothing is more satisfactory than the appearances of chloride of sodium and phosphates of potass or soda, of glycocholate of soda and hæmatoidin, of cholesterine, nucleating cell and epithelium.

The mode of examination was to place the outer liquid in a quinine bottle, and suspend the cleaned gall-bladder by a wire hung from a cork with which the bottle is closed. The bladder holding the bile was allowed to remain in the liquid 30 to 48 hours, at a temperature of 70°, then taken out, wiped dry on the outside, and placed in another vessel similarly fitted up, containing a second liquid; from this, after 30 hours, it was again removed, wiped, and placed in a third, and subsequently in a fourth liquid. I have used alcohol (86 per cent), ether, chloroform, and coal oil as the fluids best adapted. In this way, by suspensions for periods varying from 24 to 48 hours, I have separated all of the component principles in the supernatant bile (when it is allowed to rest), except the mass of epithelium, granular corpuscles, and mucous corpuscles, which do not osmose into alcohol.

The alcoholic liquid, after dialysis of a few hours, assumes an amber-yellow color, which sometimes deepens to a brown if the amorphous coloring matter of the bile be abundant. The ether and chloroform liquids are usually of a yellow-green tint, from the amount of yellow coloring matter held in suspension.

The ethereal liquid contained yellow pigment granules in great abundance, with some fatty globules, margarate soda, mucous corpuscles, and a few crystals of hæmatoidin.

When alcohol is used as the first liquid, the great portion of the liquid fatty matter passes into it, and if fresh alcohol be used to replace that deeply colored, almost the whole of the fat globules may be removed; in this way, separation between the pigment cells and fat globules may, to a great extent, be accomplished. As the bile is a fluid easily decomposed by exposure to air, the ordinary experimental dialyzer of parchment paper was not employed, the gall-bladder allows permeation to proceed in a very rapid manner, and to a considerable extent, and by its use the transfer of the liquid is avoided, the change from the condition when removed from the body a few hours after death is very slight. I therefore used the bladder without any transfer of its contents. I may state here that in the examination of the dialyzed liquids, oxidation is likely to occur, but this may be greatly, if not wholly, prevented, by the addition of creasote to the liquids. I have in this way preserved these liquids for three weeks in the *statu quo* they were in at the commencement of the experiment. Creasote has been recommended by Dr. Beale for this purpose in the examinations of the urine. I recommend it in all examinations of bile.

Having mentioned so much of the mode of examination, I will as briefly as possible state the results under the various heads of investigation.

*Quantity of Bile in Gall-Bladder.*—In ten specimens examined the greatest amount was 28 drachms, and the least 4 (four) drachms; six were above 12 drachms. Those which distended the gall-bladder the most had an unusual amount of common salt present.

*Density.*—Varied from 1020 in least amount to 1010 in that amounting to 3½ oz. As the bile is a very heterogeneous liquid, containing matters in solution and in suspension, the specific gravity gives no real information of the true disposition and mode of escape of the solid matter. When fresh bile is allowed to stand for six hours it separates into two portions—a clear, dark yellow-green liquid, and a substratum of a deep yellow tint and syrupy consistence; by separating these two layers by decantation and evaporation of both in a water bath until no further loss is appreciable, an approximate result of the relative anount of solid matter in each stratum may be obtained. I examined, in this manner, the bile amounting to 28 drachms:—

| | | |
|---|---|---|
| The solid matters in supernatant fluid | = | 6.25 per cent. |
| " " in syrupy substratum | = | 4.15 " |
| Total solid matters in bile | = | 10.40 " |

*Constitution of these Liquids.*—The two strata alluded to yielded very different results when examined. The clear fluid has a simple composition, faintly alkaline, albumen in solution not precipitable by heat alone, but by the addition of nitric acid along with or after being boiled; the liquid becomes pinkish when heated; it contains all the soluble salts of the bile. The lower stratum contained all the organic and organized substances mentioned further on. Columnar epithelium is the only substance common to both strata.

*Proximate Elements of Bile.*—The results of repeated examinations of these specimens, fluid and deposit, both under the microscope, as well as by chemical tests, has led to the following list of substances being observed as constituents:—

1. Epithelium.

2. Mucous Corpuscles.

3. Pigment Cells, yellow or greenish.

4. Brown coloring matter, resinoid and amorphous.

5. Cholesterin.

6. Fatty Globules.

7. Common Salt.

8. Carbonic and Phosphate Soda (neutral) and Potass.

9. Glycocholate Soda.

10. Margarate Soda.

11. Hæmatoidin.

12. Hippuric Acid.

13. Albumen.

It was not, of course, possible to determine the presence of all these in each specimen at first, as very many of the substances, from eight to twelve, exist in very different quantity, and if not looked for closely are apt to escape observation altogether. Under the microscope what is usually seen at first are the two varieties of pigmentary matter, the cholesterin and fat globules; the common salt will be found round the margins of the drop under observation, as beautiful Maltese crosses or dagger forms in the natural fluid; in the alcoholic extract the dodecahedron and cube forms of salt are very common.

*Cholesterin and Fats.*—The alcoholic liquid contains some epithelium and mucous corpuscles and abundant fatty globules. The latter is the chief constituent in alcohol. When this liquid stands over one day crystals of cholesterin appear; these are not regular in form, having the angles wanting; regular crystals may be obtained by re-solutions and cooling; the other fatty matters always appear as globules, clear and transparent, being much larger than the yellow-green pigment cells. I have not observed any further change of fats in the alcoholic fluid, although in ether (when cholesterin has been separated by the alcohol) silken tufts or sheafs of margarate soda are recognizable. I may remark that in all my examinations of recent bile, I have never found cholesterin as an ingredient; but when the sample in the field has stood a few hours, or if the bile itself be exposed while recent to the air, cholesterin is then found in small broken plates, similar to what may be removed from the substance of the liver by crushing a few cells and examining under the field.

The cholesterin of bile is either held in solution by the other fatty matter, or it is as yet undecomposed and a constituent of the original fat—one of the steps in the decomposition of which is to form cholesterin. However this latter may be, it is not an evident constituent of fresh bile; when the alkaline nature of the liquid from any cause is diminished, then this substance commences to appear. We can at any time produce it by adding a drop of acid (acetic or mineral) either to bile or to the crushed bile cells, when the fatty matter will be broken up and one of the resulting products is cholesterin. I do not mean to assert that cholesterin is not present, but simply not present in a free state—it is held in solution probably by the more liquid fats; for inasmuch as cholesterin is not a formation of the liver itself, being the product of decomposition of nerve matter in the blood, and merely separated in the liver—as it is sometimes in serous fluids not sufficiently alkaline—it is reasonable to conclude that its appearance in bile which has been exposed to the air or otherwise oxidized, is an evidence of its pre-existence in a soluble form.

*Yellow Coloring Matter.*—This substance dialyzes the most readily of all ingredients, passing into all of the liquids in the outer vessel; freely into alcohol, more so into ether, chloroform, and coal oil; the chloroform liquid is the most convenient for examination, but coal oil preserves the matter unchanged. The liquids have all a more or less green tint, the ethereal liquid being the deepest. Fatty matter always accompanies the coloring matter.

When examined by a power from 300 to 400 times, the structure of this substance is evident; cells globular, deep yellow color in mass, aggregated in cloudy clusters, the globules having apparently granular contents; these however are fatty matters, as treatment by alkali and acids shows, and this is the reason why it is impossible to separate by dialyses the fatty globules from the yellow-green matter. These globules become of a deeper green on addition of a little nitric or other mineral acid.

It is not further affected by nitric acid, and does not yield the iridescent tints which bile matter affords. It is not soluble in alcohol or ether, but by long contact with these liquids the cell wall becomes broken or collapsed and the fatty matter dissolves in the alcohol or ether; perhaps this is effected by osmose through the cell wall. Alkaline solutions also separate the oil, but in this case the cell wall is dissolved, and the liquid assumes a slightly red tint, showing the presence of a protein compound in the cell wall. The yellow pigment is then a globular corpuscle having an albuminous pellicle and fatty substance in the interior. I am unable to state whether the color belongs to the wall or the central contents.

Lehmann states that this pigment is amorphous and colored red by acetic acid. In every instance I have assured myself of its cellular character, nor have I observed its being reddened by acetic acid.

*Oil Globules.*—Minute oil corpuscles may be seen in great abundance in the ethereal and alcoholic liquid dialyze; of all liquids alcohol appears to favor the dialysis most apparently. As there are no free oil corpuscles in bile, its appearance on dialysis is due to the liquid acting on the yellow globules, and causing the fat to osmose; the greater portion of the fat, if not all, comes from the yellow pigment cells; globules of fat are also found attached to and escaping from the surface of cholesterin crystals, if the latter be treated by dilute sulphuric or chlorhydric acids; these in a little time disappear, and thin prismatic crystals with flat summits are formed in the vicinity. By the actions of these acids on the pigment cells tufts of silky needles of wheat-sheaf form occur scattered through the cloud of pigment.

Margarates are the fats of the pigment cell, while that obseved to escape from the cholesterine is a glycocholate; this is proved by treating the field with strong sulphuric acid, when this glycocholic acid is broken up and an occasional hemihedral crystal of *glycin* is formed.

This (distinctive) separation of the two fats must not be looked upon as existing naturally. The cholesterin must have been previously in a fluid form to be secreted, and must have passed through the hepatic cell and most likely through the pigment cells. All the fatty acids must originally have been associated and some of them conjugated.

On the treatment of these fats with acid I have never observed any form like the crystal of *taurine* or of taurocholic acid, nor any substance having the reactions proper to that acid; from my own observations I conclude it is not present in human bile, and I am not aware of any competent authority having asserted its presence. Glycocholic acid is, I believe, the true biliary acid in the human subject, and is occasionally found uncombined in bile freshly removed from the recently dead.

Dalton asserts that in human bile there is no crystallizable substance; if by this he means bile in the living subject it may be so, otherwise it is totally at variance with what I have repeatedly observed, so frequently that I do not remember one example in which I did not find it in the dialyzed liquid, where the liquid had been kept for a few days. Among the green pigment corpuscles small prismatic crystals begin to appear—four sided—with flat summits rarely overlapping, each well defined throughout; they are not seen at the same time with the pigment cell, but when the mill head screw is turned slightly, so as to approximate the stage, they then become quite distinct; they average 1-100 inches long, and at first view many appear to have the summits bevelled or dihedral, but careful observation will correct this deceptive appearance; they are always found most abundant with the greenish pigment; untreated by reagents they preserve their form for a considerable time; under the influence of acids they disappear, and as stated before, an occasional small crystal of glycin appears with a few drops of oily matter around it.

*Sources of the Morphotic Elements.*—No one who has examined the hepatic cell filled with bile, and also the pigment globules of the bile itself, can hesitate in recognizing the latter as the corpuscles of the hepatic cell; they are exactly alike in form, size, color, and consistence; they both yield fatty matter as well as coloring material, and both by aeration pass from yellow to green; both are slightly soluble in alcohol, more abundantly in ether, chloroform, and coal oil, and they do not undergo any other alteration by nitric acid in the cold than to produce the green tint. I assume it, therefore, that they are one and the same body, and that the contents of the hepatic cells are thus emptied out and delivered by the efferent ducts into the gall-bladder.

Besides these corpuscles, nuclei are seen in abundance loose among the clouds of pigment and the crystals of cholesterin; these are of larger size than could well be accommodated within the epithelial cells, and indeed these latter show little tendency to change the nucleus, being distinctly recognizable at the attached or smaller extremity; the larger free nuclei, therefore, belong to the hepatic cells, from which they have escaped along with the pigment cells; the cholesterin and other fats also escaping separate in a crystalline form.

From this it would appear that the whole contents of the hepatic cell is carried away; what, then, becomes of the cell wall? We must look upon the *brown* coloring matter as a residue of the cell wall, an altered protein substance, soluble in alkalies, not soluble in acids, assuming a violet, blue, and finally red-yellow tint by nitric acid, and showing all of the reactions by color which the bile is known to do with reagents; the play of colors of the bile is not, however, monopolized by this body, but is also given by the unaltered and partially disintegrated columnar epitelium floating in the liquid. These can by the microscope be distinctly seen to become purple and blue on the addition of niric acid.

*Brown Coloring Matter.*—This substance is readily recognized under the microscope, as appearing in masses of various sizes, rarely exceeding that of a blood corpuscle; opaque, except at the edges, where it is of an amber tint, without any trace of structure; not altered by acetic or dilute acids; soluble in alkaline solutions by warmth, and by nitric acid moderately strong, which forms an amber-yellow solution with it; producing previously the various tints of color, blue, red, and brown; the dry mass, heated on platinum foil, swells up and fuses, and in a close tube gives off ammoniacal vapors. It, at first, by the reaction, might be mistaken for a resinous substance, but is a true albuminous or protein compound in various degrees of degredation. Under certain conditions the decomposition results in the production of crystals of *hœmatoidin;* at least this latter substance has been found in situations where no other nitrogenous substance was present to produce it. The appearance of hæmatoidin has, among other considerations, led physiologists to describe this brown substance as arising from effete blood corpuscles, separated from the portal blood by the cells, and carried down by the efferent vessels; but after repeated examination, I have never found any appearances justifying this belief; this brown substance is *not found in the hepatic cells nor in the substance of the liver;* it is in the *bile* of the ducts or the gall-bladder that it is detected, and bears all the marks of being the cell wall of old and broken-down hepatic cells whose contents have previously been removed.

*Hippuric Acid—Hœmatoidin.*—These substances I have met with, one example of each, in the twenty specimens examined; I shall not offer any considerations here about their appearance.

This mode of examining the bile by dialysis and microscopic examination of the dialyzed liquids must, if supported by other observers, lead us to reject the present view of the functions of the liver as a secretion, and of the nature and sources of the bile. We must adopt the view that the bile is simply an excretion, and its formation an act of depuration of the liver, whereby it relieves itself of its own effete parts. This is the view of Dr. Draper when he writes:*—"I therefore regard the bile as an excretion of materials which are decomposing and ready to be removed from the system." But he and others suppose that the waste materials come from the *cells of the blood*, and that the blood is thus purified by the removal of degraded blood

* Human Physiology, New York, 1856.

cells in the liver. I do not say this does not occur; but it forms no appreciable amount in the contents of the bile. It is not the blood cells, but the *liver cells* which form the solid matter of the bile; the hepatic cells, their contents, and the degraded cell wall form the great bulk of the morphotic elements; columnar epithelium, in all stages from integrity to decay, mucous corpuscles, and perhaps a little tessellated epithelium, formed the remainder; rare is it to find a trace of a blood cell in any degree of decomposition. It is not from blood cells, then, nor from venous blood directly that the bile is produced, but from a *wearing down of the tissue of the liver*, removing the hepatic cells, in a ruptured and worn-out state, by the only channel through which solid matter could be removed; that is, by the bile ducts. If it be true, as put forth by some, that the open mouths of the ducts directly touch the hepatic cells without any intervening membrane, then the mode of escape is apparent; the cell nearest the extremity of the duct ruptures, and pours its contents down into the latter, and thus leaves a space for other cells in similar effete condition to repeat the process.

If the bile, then, is but an excretion, what is the function of the liver? The explanation which I have given of the constitution of bile, and its probable origin, does not alter in any degree our notions of the action of the liver, which is to purify the portal blood by removing from it the cholesterin, fatty acids, and pigments by means of the hepatic cells, and also to separate sugar to be restored again to the blood.

In separating the fatty matters it would appear that the structure of the liver itself suffers, and requires to be removed, and it may be that this removal of the hepatic cell, after they have fulfilled their secretory functions to the utmost, by the bile *is the healthy process*, and that fatty degeneration of the liver may consist, not merely in an increased growth of hepatic cells, and their granular contents becoming loaded with oil, but in the circumstance that the cells are not removed as fast as produced; that by some altered condition of the cell wall it is not ruptured and the cell contents delivered into the bile ducts; this is a point to be determined by future observation alone.

The following conclusions are, I think, fairly deducible from the above:—

1. The bile is an albumino-serous liquid, holding diffused organic and organized matter, whose structure and origin may be traced by microscopic investigation.

2. The organized matters are derived from the *hepatic cells*—chiefly its inner granular matter—and from the epithelium lining the bile duct.

3. The fats of the bile are the fats of the bile cell derived from it, and have suffered no alteration, except their escape from inside the cell.

4. The bile contains two distinct coloring matters, the yellow and the brown; both of cellular origin, the latter only in a state of decay.

5. The yellow coloring matter of the bile is an organized body—a cell containing cloudy granular contents, which furnish fatty matter by decomposition; the fatty acids being the margaric and glycocholic, associated with cholesterin and soda; these yellow corpuscles are the yellow corpuscles of the bile cell.

6. The brown coloring matter is a protein compound, an albuminous substance undergoing decomposition, and is probably the remains of the cell wall of the hepatic cell whose contents constitute the matter described in No. 5. It is this brown matter which, by treatment, with nitric acid, yields the play of colors used as a test for the presence of bile.

7. The bile contains both the *materials* removed from the portal blood and the *anatomical structures* by which the removal was effected; the latter in a stage of retrogressive metamorphosis.

8. The bile is an excretion—a depuration of the liver directly, of the blood remotely.

As all of the specimens of bile alluded to in this paper were taken from diseased subjects, it may be objected to any inferences drawn that the bile does not represent that in health; as, however, the object of this paper is not so much to describe the chemical characters of normal bile as to show in what form the substances are actually found in human bile, the objection can have but little force in this place.

